# From GWAS to genome sequencing: complementary approaches to identify melanoma predisposition genes

**DOI:** 10.1186/1897-4287-10-S2-A46

**Published:** 2012-04-12

**Authors:** S MacGregor, KM Brown, M Stark, M Gartside, S Woods, V Bonazzi, L Aoude, K Dutton-Regester, S Tyagi, J Liu, DL Duffy, J Palmer, A Cust, H Schmid, J Symmons, E Holland, C Agha-Hamilton, K Holohan, D Youngkin, E Gillanders, MA Jenkins, J Kelly, DC Whiteman, R Kefford, G Giles, B Armstrong, J Aitken, J Hopper, G Montgomery, C Schmidt, JM Trent, NG Martin, GJ Mann, NK Hayward

**Affiliations:** 1 Queensland Institute of Medical Research, Brisbane, QLD, Australia; 2 Translational Genomics Research Institute, Phoenix, AZ, USA; 3 University of Melbourne, Melbourne, VIC, Australia; 4 Westmead Institute for Cancer Research, University of Sydney, Sydney, NSW, Australia; 5 Cancer Council Victoria, Melbourne, VIC, Australia; 6 Alfred Hospital, Melbourne, Australia; 7 Cancer Council Queensland, Brisbane, QLD, Australia; 8 University of Sydney, Sydney, Australia; 9 National Cancer Institute, Rockville, MD, USA

## 

Family and twin studies indicate that melanoma susceptibility has a strong genetic component. Very rarely, melanoma runs in families in which there is an inherited mutation in a single ‘high penetrance’ gene, but in the general population melanoma susceptibility is thought to be governed by variation in a series of ‘low penetrance’ genes. We sought to identify new melanoma risk genes of both classes by conducting an Australian genome-wide association study (GWAS) of ~2200 melanoma cases and ~4300 matched controls (from the AMFS and Q-MEGA studies), in parallel with whole-genome sequencing of cases from densely affected melanoma families with follow up genotyping of interesting variants in the GWAS sample and other highly case-loaded melanoma families.

Genotyping of the GWAS sample was carried out using Illumina Hap610K or OMNI 1M arrays. All 25 SNPs that reached genome-wide statistical significance (i.e. p<5 x 10^-8^) map to chromosomal regions/genes previously associated with melanoma (e.g. MC1R, ASIP, OCA2, MTAP/CDKN2A and SLC45A2). However, two other genomic regions had multiple adjacent SNPs with low p values. These were the focus of a replication study using melanoma GWAS data generated by groups from the MD Anderson Cancer Center and the International Melanoma Genetics Consortium (GenoMEL). Both independent GWAS data sets support the original Australian findings and thus indicate that the PARP1 gene and another broad region on 1q (which includes SETDB1, a recently identified melanoma oncogene) are novel low penetrance melanoma risk loci.

The three known high penetrance melanoma susceptibility genes (*CDKN2A*, *CDK4* and *ARF*) account for less than half of all ‘familial’ melanoma. We sought to identify other genes responsible for susceptibility in multi-case melanoma families using a next-generation sequencing approach. Families were chosen on the basis that they did not have a mutation in *CDKN2A*, *CDK4* or *ARF*; and had at least 5 melanoma cases. Whole-genome or exome sequencing was carried out on X cases from Y families. No convincing evidence has yet been obtained to support the identification of a new familial melanoma gene, however, follow up genotyping of several novel SNPs in the GWAS sample showed that a non-synonymous variant in the MITF gene is associated with melanoma in the general population. This type of integrated approach should help accelerate the discovery of new loci that play a role in the aetiology of melanoma.

